# Towards a structured ophthalmology residency program in Oman

**DOI:** 10.4103/0974-620X.60012

**Published:** 2010

**Authors:** Abdullah Al-Mujaini

**Affiliations:** Chairman of Ophthalmology Residency Program, Oman Medical Specialty Board, Muscat, Sultanate of Oman

Starting a residency program is a huge endeavor that requires both program organization and widespread support from various groups. The Oman Medical Specialty Board (OMSB), with a mission of developing and maintaining postgraduate medical specialty education in the country and setting the professional and educational standards for the training and certification of Medical and Healthcare Professionals, set to the task of establishing an Ophthalmology Residency Program in 2006. OMSB deemed it necessary to form the Ophthalmology Residency Training Program to train personnel in this challenging surgical specialty.[1]

A task force committee was constituted in 2007 to formulate requirements and design guidelines for the residency program. The Scientific Committee for the Ophthalmology Training Program was formed in April 2009. Internal reviewers and external reviewers from USA and Saudi Arabia then evaluated the proposed residency program and the available infrastructure and manpower. To the utmost happiness of all concerned, they were very positive about the possibility of commencing the program through the involvement of two tertiary ophthalmology health care centers in Oman (Sultan Qaboos University Hospital and Al-Nahda Hospital). However, they proposed incorporating a few major changes in the existing system to facilitate resident training. With the departments of the training centers initiating steps to make those changes, the two centers were accredited by the OMSB. It was not until August 29, 2009, when four residents started for the first time their Ophthalmology Residency Training under the umbrella of OMSB, paving the way for a new era in specialty training in the country.[2]

The Ophthalmology Residency Training Program will incorporate the six general competencies for residents, as endorsed by the Accreditation Council for Graduate Medical Education, considered relevant to the practice of ophthalmology: Patient care, medical knowledge, practice-based learning and improvement, interpersonal and communication skills, professionalism, and system-based practice. The program aims to produce competent general ophthalmologists that will have knowledge both in general ophthalmology and in subspecialties. They will have excellent clinical and surgical skills, awareness of medical ethics, professional values and attitudes, and will have the capability of conducting teaching and research activities. We believe the graduates of this program will acquire the skills to be utilized for the benefit of patients and the education of the next generation of ophthalmologists in the country.

There will be many obstacles, but I’m quite confident that the new generation of qualified ophthalmologists, who have received their training outside the country, will be able to carry the flame and pass it on successfully.

## References

[1] (2007). Curriculum manual for the residency training programs in ophthalmology. Saudi Commission for Health Specialties. 1st revision.

[2] (2008). Ophthalmology specialty training program curriculum. Oman: Oman Medical Specialty Board.

